# Molecular Characterization and Interaction between Human VEGF-D and VEGFR-3

**DOI:** 10.4014/jmb.2409.09060

**Published:** 2024-11-20

**Authors:** Chae Eun Seo, Han Na Lee, Mi Suk Jeong, Se Bok Jang

**Affiliations:** 1 Department of Molecular Biology, College of Natural Sciences, Pusan National University, Busan 46241, Republic of Korea; 2 Insitute of Systems Biology, Pusan National University, Busan 46241, Republic of Korea

**Keywords:** Angiogenesis, lymphangiogenesis, VEGF-D, VEGFR-3, mutation, interaction

## Abstract

Angiogenesis and lymphangiogenesis are some of the routes that cause metastasis. Vascular Endothelial Growth Factors (VEGFs) stimulate angiogenesis and lymphangiogenesis through VEGF receptors. Especially, VEGF-D and its receptor, VEGFR-3, play a pivotal role in regulating cellular processes such as survival, proliferation, and migration, thereby influencing lymphangiogenesis. The aim of this research is to clarify the molecular characteristics of VEGF-D and VEGFR-3 proteins and identify the key residues that are essential for the interaction between VEGF-D and VEGFR-3. Experiments, including size exclusion chromatography and GST pull-down assay analysis, reveal that specific residues, particularly D103 and Q110, are essential for VEGF-D/VEGFR-3 binding. Mutations in these residues induce structural alterations, resulting in reduced binding affinity and impaired activation of VEGFR-3. Moreover, this study suggests that a synthesized peptide, designed based on key residues of VEGF-D involved in binding to VEGFR-3, may act as a metastasis suppressor by competitively inhibiting the interaction between VEGF-D and VEGFR-3. Understanding these molecular interactions is expected to have significant potential to develop therapeutic peptides that can inhibit cancer cell-induced lymphangiogenesis and resolve metastasis via lymphangiogenesis across various cancer types.

## Introduction

Cancer can spread to a different part of body than where it started; this phenomenon is called “metastasis.” Angiogenesis and lymphangiogenesis are among the routes that cause metastasis [1, 2]. Several studies on angiogenesis and angiogenic factors have been performed, and several drugs based on these have been developed to target them, suppressing metastasis. Vascular endothelial growth factors (VEGFs) are signal proteins that trigger angiogenesis and lymph angiogenesis with VEGF receptors (VEGFRs). In human, the VEGF family is composed of VEGF-A, VEGF-B, VEGF-C, VEGF-D, and placenta growth factor (PlGF) [5]. VEGF-A stimulates angiogenesis by interacting with VEGFR-1 and VEGFR-2. VEGF-B and PlGF only bind to VEGFR-1 for angiogenesis. VEGF-C and VEGF-D can induce angiogenesis and lymphangiogenesis by binding to VEGFR-2 and VEGFR-3 respectively [6].

In various studies, it has been reported that the formation of a new lymph vessel near solid tumors correlates with lymphatic metastasis in breast cancer, lung cancer and thyroid cancer [7-9]. The central molecules during the growth of the lymphatic system are VEGF-C and VEGF-D [10, 11]. When they activate VEGFR-3, its signal not only induces cell proliferation, migration, and invasion but also promotes lymphangiogenesis [12]. Prior studies have discovered a correlation between VEGF-C/ -D and lymph node metastasis in several human cancers. Moreover, there are experimental results that employ VEGF-D-specific antibodies in a VEGF-D-positive tumor model that can block lymphatic metastasis [13, 14]. Because VEGF-D is related to not only lymphatic metastasis but also other diseases such as pulmonary diseases [15], urologic diseases [16, 17], and cardiovascular diseases [18], there have been several efforts to develop reagent-targeting VEGF-D. However, unlike VEGF-C, there are insufficient reports on the interactions between VEGF-D and VEGFR-3. During development, the VEGF-D gene is expressed in various tissues and, in adults, is remarkably expressed in lung and skin tissues [11, 19]. Furthermore, the secretion of VEGF-D correlates with inflammation and cancer [20, 21].

The enzymes include plasmin, kallikrein related peptidase 3 (KLK3), cathepsin D, and thrombin. Because these enzymes target different regions, various spliced forms are generated [22]. Among the fragments, the most dominant thing is residue 89-205 which is also called major form, and residue 100-205 is the minor form. The final form for activation is processed as the dimer form. Although both of these proteins are mature version of VEGF-D, the minor form cannot activate VEGFR-3 [23]. The structure of VEGF-D has been partially revealed (PDB code 2XV7), and it includes residue 91-194.

VEGFRs are tyrosine kinase receptors that play central roles in angiogenesis and lymphangiogenesis. Specifically, VEGFR-3 signaling is related to the migration, proliferation, and survival of lymphatic endothelial cells [24]. Lymphangiogenesis via VEGFR-3 enhances the mobility and invasive capabilities of cancer cells, thereby promoting metastasis.

The initial expression of VEGFR-3 occurs during the early phase of embryonic development in both blood endothelial cells and lymphatic endothelial cells. In adults, VEGFR-3 is specifically expressed in lymphatic endothelial cells [25]. However, the expression level of VEGFR-3 in peritumoral or inflammation areas is relatively high compared to that in normal lymphatic vessels [26-28]. Because of the correlation between VEGFR-3 and cancer cell metastasis, such a relatively higher expression level can lead to a decrease in the patient's survival rate. Like many other receptors, VEGFR-3 also consists of extracellular, transmembrane, juxtamembrane, and protein kinase domains. The extracellular domain contains seven Ig domains [29]. VEGF-D binding occurs mainly in domain 1, and domains 2 and 3 increase the affinity for VEGF-D [23]. However, it remains unclear where the direct interaction residues are. So far, the structure of VEGFR-3 extracellular domains D4-5 (PDB code 4BSJ) and structure of VEGFR-3 extracellular domains D1-2 in complex with VEGF-C (PDB code 4BSK) have been revealed [30].

As is already known, there are closer similarities between VEGF-C and VEGF-D than between other members within the VEGF family. In a paper that studied the binding of VEGF-C and VEGFR-3, the researchers analyzed that the key residue in this interaction is the D123 and Q130 of VEGF-C [30]. When aligning VEGF-C and VEGF-D, D123 and Q130 in VEGF-C corresponds to D103 and Q110 in VEGF-D [11]. Based on their structural similarity, it can be assumed that D103 and Q110 of VEGF-D are crucial for interaction between VEGF-D and VEGFR-3. Meanwhile, another study claimed that Y94, L99, I102 and E105 are essential [31]. Based on previous studies, interrupting the binding between VEGF-D and VEGFR-3 will likely inhibit metastasis via lymphangiogenesis [32]. This study aimed to confirm which residue was more ideal for VEGR-3 binding and experiments were conducted using peptides based on D103 and Q110. It is expected that identifying significant residues assists in developing peptides specific for VEGF-D and VEGFR-3 interactions for therapeutic purposes.

## Materials and Methods

### Recombinant VEGF-D and VEGFR-3

The wild-type human VEGF-D (residue 89-205) and VEGFR-3 (residue 30-326) were inserted into the His-tagged fusion protein vector pET-28a for purification. To perform a glutathione S-transferase (GST) pull-down assay, mature VEGF-D (residue 89-205) and its mutated version were subcloned into a GST-fused protein vector pGEX-4T-1. VEGF-D and VEGFR-3 were amplified by polymerase chain reaction with synthetic oligonucleotide primers. The primer information utilized for the construction of the recombinant VEGF-D, mutated VEGF-D and VEGFR-3 plasmid is detailed in Table S1. Amplified genes and each plasmid were digested overnight at 37°C. Afterward, T4 ligase (ELPIS-BIOTECH, Republic of Korea) combined the insert and vector.

### Protein Expression of VEGF-D and VEGFR-3

Both plasmid pET28a/ VEGF-D and pET28a/ VEGFR-3 were transformed into *Escherichia coli* BL21 (DE3) for overexpression. Then, each colony was inoculated in 10 ml of Luria Bertani (LB) media with 10 μg/ml kanamycin, and grown overnight at 37°C. The overnight cultures were diluted 1:100 in 1 L of fresh LB medium with 10 μg/ml kanamycin and further incubated at 37°C. Once OD600 reached 0.5-0.6, protein expression was induced using 0.5 mM IPTG at 25°C overnight. The cells were harvested by centrifugation at 4,500 rpm for 25 min at 4°C, and the collected cell pellets were stored at -70°C. The VEGF-D and VEGFR-3 cell pellet were suspended in lysis buffers, respectively, and then cells were disrupted using a sonicator in ice. Cell lysates were centrifuged at 13,500 rpm for 45 min and then the supernatants were removed. The VEGF-D and VEGFR-3 pellets were sonicated again, and the resulting supernatants were collected by centrifugation at 13,500 rpm for 45 min.

The GST-tagged VEGF-D and its mutants were transformed into *E. coli* BL21 (DE3) for expression. Each colony was incubated in 10 ml of LB media with 100 μg/ml ampicillin and grown overnight at 37°C. These cell cultures were then added to 1 L of fresh LB medium, including 100 μg/ml ampicillin, and further incubated at 37°C until an OD600 of 0.5 was reached. Protein expression was induced by adding 0.5 mM IPTG to 1 L of LB medium and incubating overnight at 25°C. To harvest the cells, the culture media were centrifuged at 4,500 rpm for 25 min at 4°C. The supernatants were removed and the pellets were frozen at -70°C for storage. To resuspend, the pellets were sonicated in ice. After centrifugation of the cell lysates at 13,500 rpm for 45 min, the supernatants were discarded. A second round of sonication was performed with lysis buffer F, and the resulting supernatants were collected by centrifugation at 13,500 rpm for 45 min. For co-lysis, His-tagged VEGF-D and His-tagged VEGFR-3 cells were sonicated together. The cell lysates were centrifuged at 13,500 rpm for 45 min, and the resulting supernatants were removed. The pellets were then resuspended in lysis buffer and centrifuged again at 13,500 rpm for 45 min. Finally, the supernatants were collected.

### Purification of VEGF-D and VEGFR-3 Proteins

The supernatant containing His-tagged VEGF-D proteins was loaded onto a Ni-NTA column (Bio-Rad, USA) that was pre-equilibrated with buffer A [6M Urea, 50 mM HEPES (pH 7.7), 200 mM NaCl, 10% glycerol]. Then, the column was washed with buffer A added to 20 mM imidazole. The absorbed proteins were eluted with buffer A containing 100/200 mM imidazole at a flow rate of 5 ml/min. The purification process was analyzed by 15% SDS-PAGE and the fractions containing VEGF-D were concentrated using Macrosep Advance Centrifugal Devices with 3K MWSO (Pall Co., USA) and Amicon Ultra-15 Centrifugal Filter Unit (Merck, Germany) at 3,000-3,500 rpm. For refolding, urea in the solution was reduced by buffer changing with buffer B [50 mM HEPES (pH 7.7), 200 mM NaCl, 10% glycerol].

The supernatants of His-tagged VEGFR-3 proteins were loaded onto a pre-equilibrated Ni-NTA column by buffer C [6 M Urea, 50 mM Tris-HCl (pH 7.82), 200 mM NaCl, 10% glycerol]. The column was washed with buffer B added to 10 mM imidazole and the absorbed proteins were eluted with buffer B containing 100/200 mM imidazole at a flow rate of 5 ml/min. Likewise, the purification process was analyzed by 15% SDS-PAGE and the VEGFR-3 fractions were concentrated using Macrosep Advance Centrifugal Devices with 3K MWSO (Pall Co.) and Amicon Ultra-15 Centrifugal Filter Unit (Merck) at 3,000-3,500 rpm. By buffer changing with buffer D [50 mM Tris-HCl (pH 7.82), 200 mM NaCl, 10% glycerol], urea in the solution was reduced and proteins were refolded.

In order to get high pure VEGFR-3 sample, a fast protein liquid chromatography (FPLC) was conducted. A Superdex 200 10/300 GL column was equilibrated with buffer D and then the proteins were allowed to flow the column. The protein mixtures were separated at a flow rate of 0.4 ml/min.

To pre-equilibrate a Glutathione-sepharose 4 Fast Flow column (GE Healthcare, USA), the column was washed with buffer E [6 M Urea, 50 mM Tris-HCl (pH 7.82)]. Afterward, the lysates containing GST-tagged VEGF-D or its mutated constructs were loaded onto the column. Buffer E was used to wash out the column to eliminate the unabsorbed materials and the absorbed proteins were eluted with buffer E containing 5/10 mM glutathione at a flow rate of 5 ml/min. Buffer D was used for reducing urea in the solution for protein refolding.

The supernatants of the co-lysate were loaded onto a pre-equilibrated Ni-NTA column with buffer C [6 M Urea, 50 mM Tris-HCl (pH 7.82), 200 mM NaCl, 10% glycerol]. The unbound proteins were washed away using buffer B added to 20 mM imidazole and the absorbed proteins were eluted with buffer B containing 100/200 mM imidazole at a flow rate of 5 ml/min.

### Western Blotting

For Western blotting, 15% SDS-PAGE was conducted at 90V for 3 h with the purified VEGF-D and VEGFR-3. Proteins on SDS polyacrylamide gels were transferred onto an immobilon-P transfer membrane (Millipore, USA) at 105V for 1h. The membranes were then blocked using ProNA 5X General-Block Solution (TransLab., Republic of Korea) diluted to 1X with 1X PBS with 0.1% Tween 20 (PBS-T) for 1h. Afterward, the membranes were incubated overnight at 4°C with the first antibody [His-Tag(H-3):sc-8036 diluted to 1:1000 (Santa Cruz Biotechnology, USA), and GST(B-14) diluted to 1:2000 (Santa Cruz Biotechnology)]. Then, the membranes were washed with PBS-T and incubated in the second antibody [Anti-mouse IgG 1:2000 (Cell Signaling Technology, USA)] for 1 h. Signal detection was performed to visualize the bands using Western ECL Kit (LPS solution, Republic of Korea). These results were assessed using FUSION FX (Vilber, France).

### Glutathione S-Transferase (GST) and His-Tagged Pull-Down Assay

For the GST pull-down assay, 50 μg of GST and GST tagged VEGF-D and mutated constructs were used. Proteins bound to Glutathione Sepharose 4 Fast Flow beads (Amersham Biosciences, UK) were incubated with 50 μg of His-VEGFR-3 and 1X PBS in ice for 10 min on a rocking platform. After rocking incubation, mixtures were kept in ice overnight. Subsequently, the resin was washed three times with 1X PBS at 3,000 rpm for 5 min. The binding proteins were eluted with an elution buffer [50 mM Tris-HCl (pH 7.82) and 30 mM glutathione] and 15%SDS-PAGE was conducted. Subsequently, Western blotting was carried out with anti-His and anti-GST antibodies.

### Circular Dichroism (CD) Spectroscopy

CD spectroscopy was used for estimating protein secondary structure of proteins. Wild-type VEGF-D, VEGFR-3 and variant proteins (I102A/ E105A and D103A / Q110) were examined by CD using a JASCO J-1500 CD spectrometer with a 1 mm pathlength cell. Protein concentration was 0.5 mg/ml in 50 mM NaCl and 50 mM HEPES (pH 7.7) (VEGF-D) or 50 mM Tris-HCl (pH 7.82) (VEGFR-3 and variant proteins). Samples were scanned from 260 to 190 nm (Far UV) and spectra were acquired every 0.1 nm with a 1 s averaging time per point and a 1 nm bandpass. To decrease noise, each spectrum was obtained through three scans and smoothed before structure analysis was performed. The secondary structural prediction was conducted using CDNN program.

### Surface Plasmon Resonance (SPR) Analysis

Binding analysis was performed on a Biacore T100 biosensor (GE Healthcare, Sweden) at 25°C using [50 mM HEPES (pH 7.7), 50 mM NaCl] or [50 mM Tris-HCl (7.82), 50 mM NaCl] as a running buffer. The VEGFR-3 protein was immobilized on a CM5 sensor chip (GE Healthcare) at a concentration corresponding to 2,300 response units using an amine coupling method. For binding analysis, serially diluted VEGF-D and mutation protein were diluted in [50 mM HEPES (pH 7.7), 50 mM NaCl] or [50 mM Tris-HCl (7.82), 50 mM NaCl] and injected over the chip at a ﬂow rate of 10 μl/min. The regeneration of the immobilized ligand was performed with 50 mM NaCl, before every sample injection.

### Size-Exclusion Chromatography (SEC)

SEC was used to check recombinant VEGF-D/VEGFR-3 binding. A Superdex 200 10/300 GL column was equilibrated with a buffer [50 mM Tris-HCl (pH 7.82), 200 mM NaCl and 0.1% glycerol], and then the protein sample derived from co-lysis was loaded onto column. The concentration of the sample used was 17 mg/ml. After sample injection, the experiment was performed at a flow rate of 0.4 ml/min and the result was analyzed using a fraction graph and SDS-PAGE.

### Prediction of the Secondary Structure and Modeling of the Complex Structures

The reference proteins of VEGF-D (PDB code 2XV7; residues 91-194), VEGFR-3 (PDB code 4BSK; residues 28-224) and VEGF-C (PDB code 4BSK; residues 115-212) were used. The three-dimensional prediction models of the VEGF-D dimer and the VEGF-D/VEGFR-3 complex were generated using Z-dock [34]. The superimposed figure was created using PyMOL. The secondary structure of VEGF-D, VEGFR-3 and VEGF-C were predicted by Jalview.

### Peptide Synthesis

The peptide of VEGF-D was designed based on alignment with VEGF-C residues that were reported to be essential to the interaction between VEGF-C and VEGFR-3. The peptide was synthesized using Fmoc solid-phase peptide synthesis (Peptron Inc., Republic of Korea) and purified up to 95% purity by reverse-phase high-performance liquid chromatography. Subsequently, liquid chromatography/mass spectrometry (LC/MS; HP 100 Series; Agilent Technology, USA) was employed to identify the peptide.

### Cell Culture

MDA-MB-231 (breast cancer) cells were purchased from the Korea Cell Line Bank (Korea Cell Line Bank, Republic of Korea). All cell lines were cultured in 100 nm cell culture dishes and incubated under standard condition (37°C under a humidified atmosphere containing 5% CO_2_) in RPMI-1640 media with fetal bovine serum, 100 Unit/ml penicillin (Welgene, Republic of Korea).

### Cell Viability Assay

Cell proliferation and viability were measured using the 3-(4,5-dimethylthiazol-2-yl)-2,5-diphenyltetrazolium bromide (MTT; Sigma Aldrich, USA) assay. Cells (4 × 10^4^ cells/well) for MDA-MB-231 cell lines were seeded on 48-well plates and 96-well plates. The cells were allowed to attach for 24 h after plating, and then treated with fresh media. Subsequently, they were treated with various concentrations of peptide or VEGF-D protein. After 24 h of incubation, the MTT solution was added, following which the cells remained for reaction at 37°C under 5% CO_2_ for 90 min. The optical density (OD) of each well was measured using a microplate reader (Biotek Winooski, USA) at 540 m.

### Statistical Analysis

All statistical tests were conducted using an unpaired Student’s *t*-test and one-way ANOVA, followed by Bonferroni’s correction. The statistical analyses were performed with GraphPad Prism version 8.4.2 (GraphPad Software, USA).

## Results

### Structural Characterization of VEGF-D and VEGFR-3 Protein

**Fig. 1:**
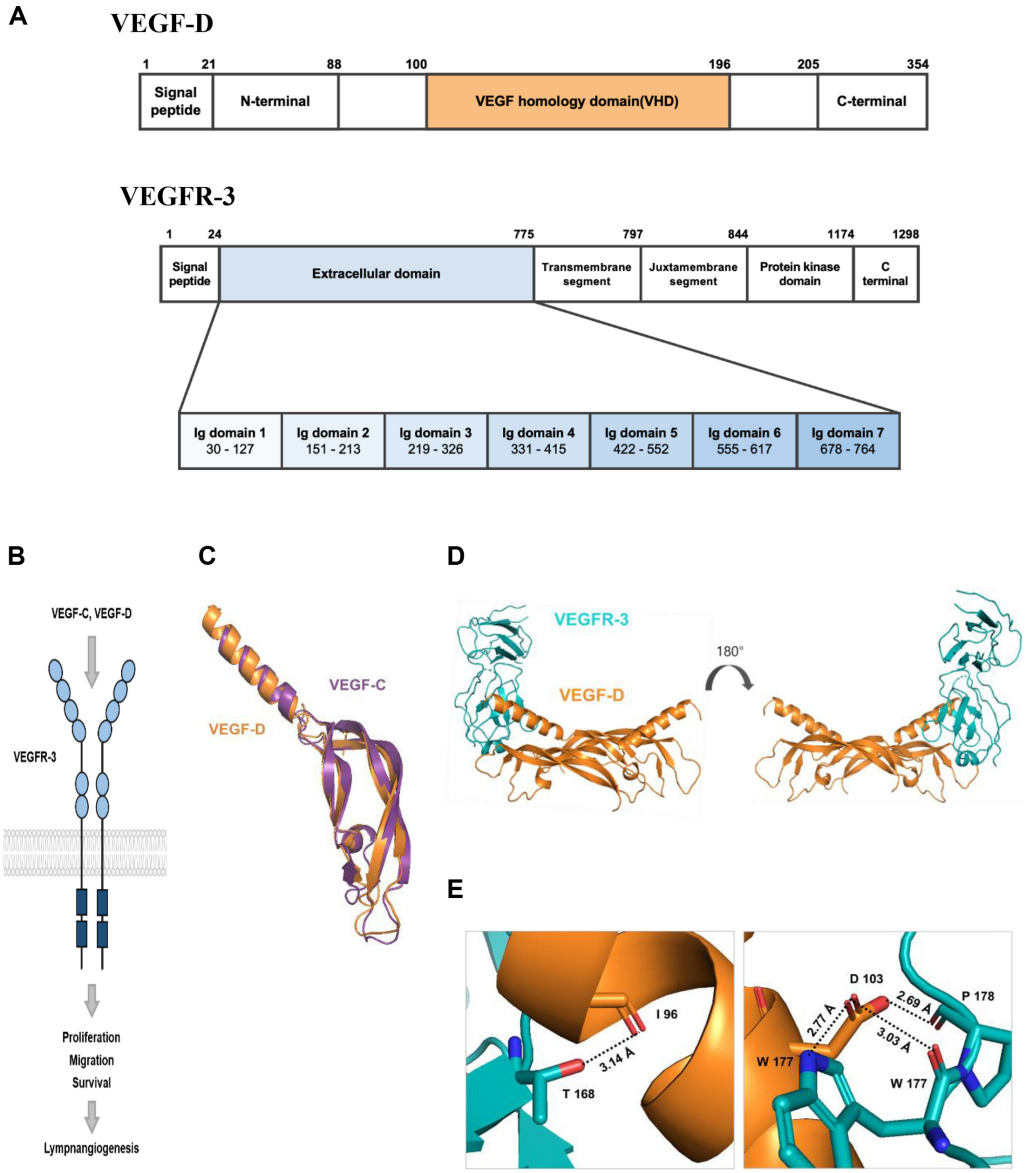
Characteristics of VEGF-D, VEGFR-3, and VEGF-C. (**A**) Schematic representation of the domain structure of full-length VEGF-D (residues 89-205) and VEGFR-3 (residues 30-326) is shown. (**B**) Schematic representation of VEGFR-3 and its ligands signaling pathway. (**C**) VEGF-D monomer (PDB code 2XV7) and VEGF-C monomer (PDB code 4BSK) are overlapped to compare their structure. (**D**) The prediction model of VEGF-D (orange and pale yellow) dimer and VEGFR-3 (cyan). VEGF-D dimer structure and VEGF-D/VEGFR-3 complex structure are generated using ZDock. To construct model, PDB code 2XV7 and 4BSK were used as a reference. (**E**) The residues indicated in the figure are presumed that are important for VEGF-D and VEGFR-3 binding.

Like other members of the VEGF family, VEGF-D also has VEGF homology domain (VHD; residues 101-196), containing the receptor binding region [11]. The VHD is located between the N- and C-terminal accessory sequences that will be removed for activation by two proteolytic cleavages. After the proteolytic process, two mature proteins are generated; the major form (89-205) and the minor form (100-205) (Fig. 1A). VEGFR-3 can be divided into extracellular domain, transmembrane segment, juxtamembrane segment, and protein kinase domain. The study also employed extracellular domains 1, 2, and 3 for VEGF-D binding to VEGFR-3 (Fig. 1A). VEGFR-3 signaling pathway is depicted. VEGFR-3 is mostly expressed on lymphatic endothelial cells. The study will identify experiment about VEGFR-3 activation with VEGF-D induces cell proliferation and survival. This signaling leads to lymphangiogenesis (Fig. 1B). The secondary structures of VEGF-D and VEGFR-3 were predicted with Jalview (Fig. S1A and S1B). The data show that the N-terminal side of VEGF-D is rich in α-helices (red tube) and the other side consists of β-sheets (green arrow). Meanwhile, VEGFR-3 is mainly comprised of β-sheets. Another lymphangiogenic factor, VEGF-C, also activates VEGFR-3 and is structurally similar to VEGF-D. Both these have a similar structure with an α-helix in front of a β-sheet, and the α-helix region within the VHD, in particular, is essential for interaction with VEGFR-3. To demonstrate the similarity of the protein sequences, we performed a sequence alignment between VEGF-C and VEGF-D, resulting in an alignment of 34% (Fig. S2A). Similar to the secondary structure prediction of VEGF-D, VEGF-C also has an α-helix preceding the β-sheet (Fig. S2B).

The quantitative RMSD value of 1.319Å was measured after superimposing the three-dimensional structures of the VEGF-D monomer (PDB code 2XV7) and VEGF-C monomer (PDB code 4BSK) using PyMOL. Both structures are of the region corresponding to the VHD, and the high similarity of the α-helical region can be seen (Fig. 1C). A three-dimensional structure model was obtained using SWISS-MODEL (ExPASy). The orange and pale-yellow elements exhibit VEGF-D (PDB code 2XV7) dimer that generated by ZDock and cyan element exhibit VEGFR-3 (PDB code 4BSK) (Fig. 1D). The enlarged picture represents the residues expected to interact with VEGFR-3 (Fig. 1E and Table S2). Another lymphangiogenic factor, VEGF-C that also activates VEGFR-3 is structurally analogous to VEGF-D.

### The Decreased Affinity between VEGF-D and VEGFR-3 as a Result of Mutation at D103 and Q110 within VEGF-D

**Fig. 2:**
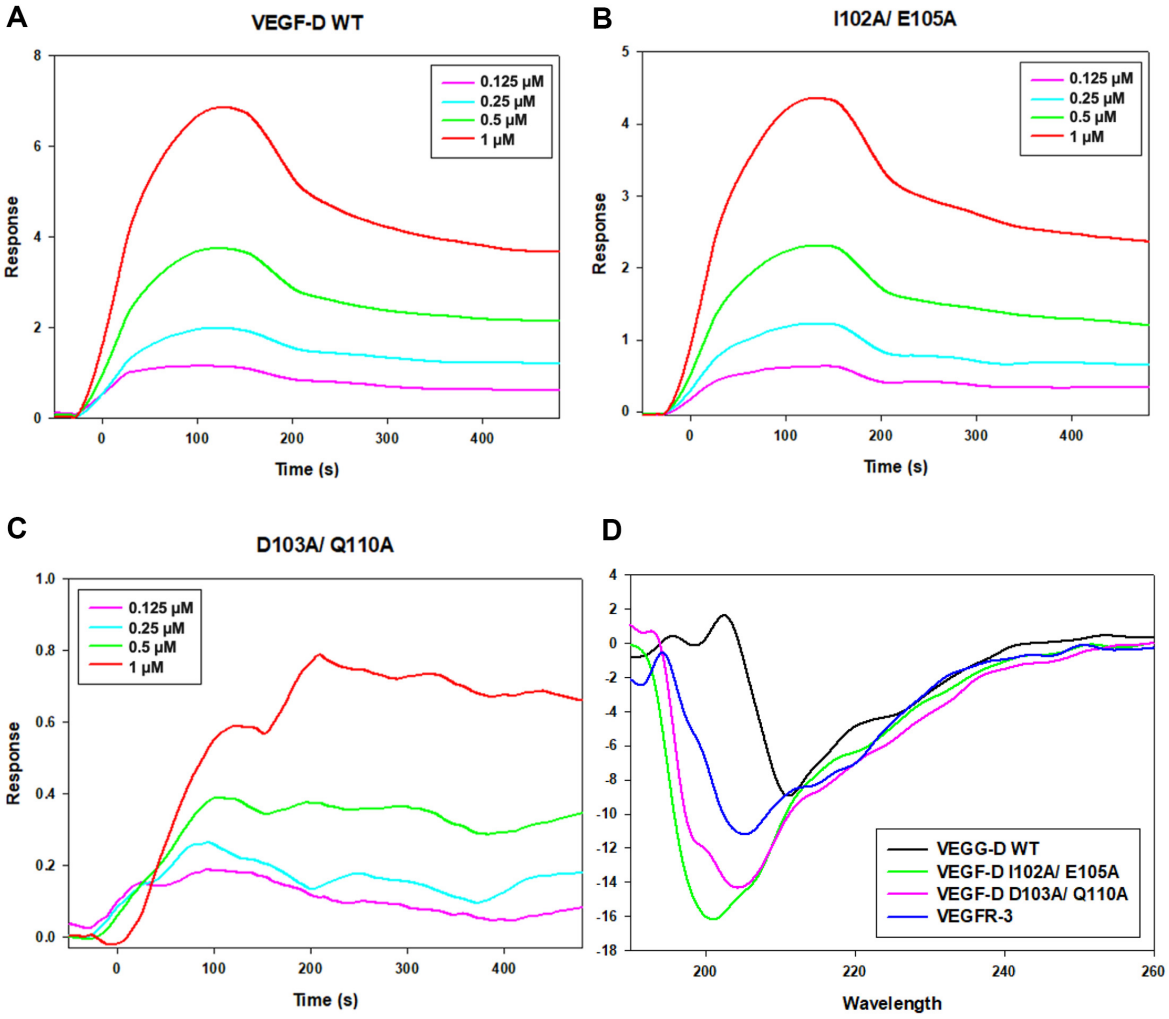
Surface plasmon resonance and circular dichroism analyses. (**A-C**) Surface plasmon resonance (SPR) analysis of wild-type and mutants (I102A/ E105A and D103A/ Q110A) of VEGF-D with VEGFR-3. Protein sensorgram for 0.125, 0.25, 0.5, 1 μM are shown. (**D**) The far-ultraviolet (UV) circular dichroism (CD) spectrum was collected from 190 to 260 nm. The CD spectrum of wild-type VEGF-D (residues 89-205), variants (I102A/E105A and D103A/Q110A) and VEGFR-3 (residues 30-326) was measured. The signals were combined into CDNN.

Several experiments were conducted to figure out the difference between the wild-type and variants of VEGF-D. To investigate differences in affinity with VEGFR-3 between wild-type, I102A/ E105A and D103A/ Q110A of VEGF-D, surface plasmon resonance (SPR) analysis was conducted (Fig. 2A-2C and Table S3). The analysis was carried out with concentrations of 0.125, 0.25, 0.5, and 1 μM. The equilibrium dissociation constant (K_D_) of wild-type, I102A/ E105A and D103A/ Q110A were calculated as 2.7 × 10^-7^ M, 9.6 × 10^-7^ M and 5.3×10^-7^ M, respectively.

Next, Circular dichroism (CD) analysis was conducted to find out structural changes in mutants (Fig. 2D and Table S4). The graph represents that the structure of I102A/ E105A and D103A/ Q110A has changed significantly in both. Moreover, it was observed that there was also a change in the detailed ratio. Secondary structure modification is also observed in both variants compared to the wild-type (Fig. 2D). The proportion of α-helix in wild-type VEGF-D is 10%, meanwhile, those in I102A/ E105A and D103A/ Q110A are 8 and 7%, respectively. The proportion of β-sheet in wild-type VEGF-D is 20% and those decrease in I102A/ E105A and D103A/ Q110A are 29 and 36%, respectively. There is no detrimental difference in the proportion of turn, whereas the proportion of random coil increases in I102A/ E105A to 58% and in D103A/ Q110A to 54% in comparison with 64% of wild-type.

**Fig. 3:**
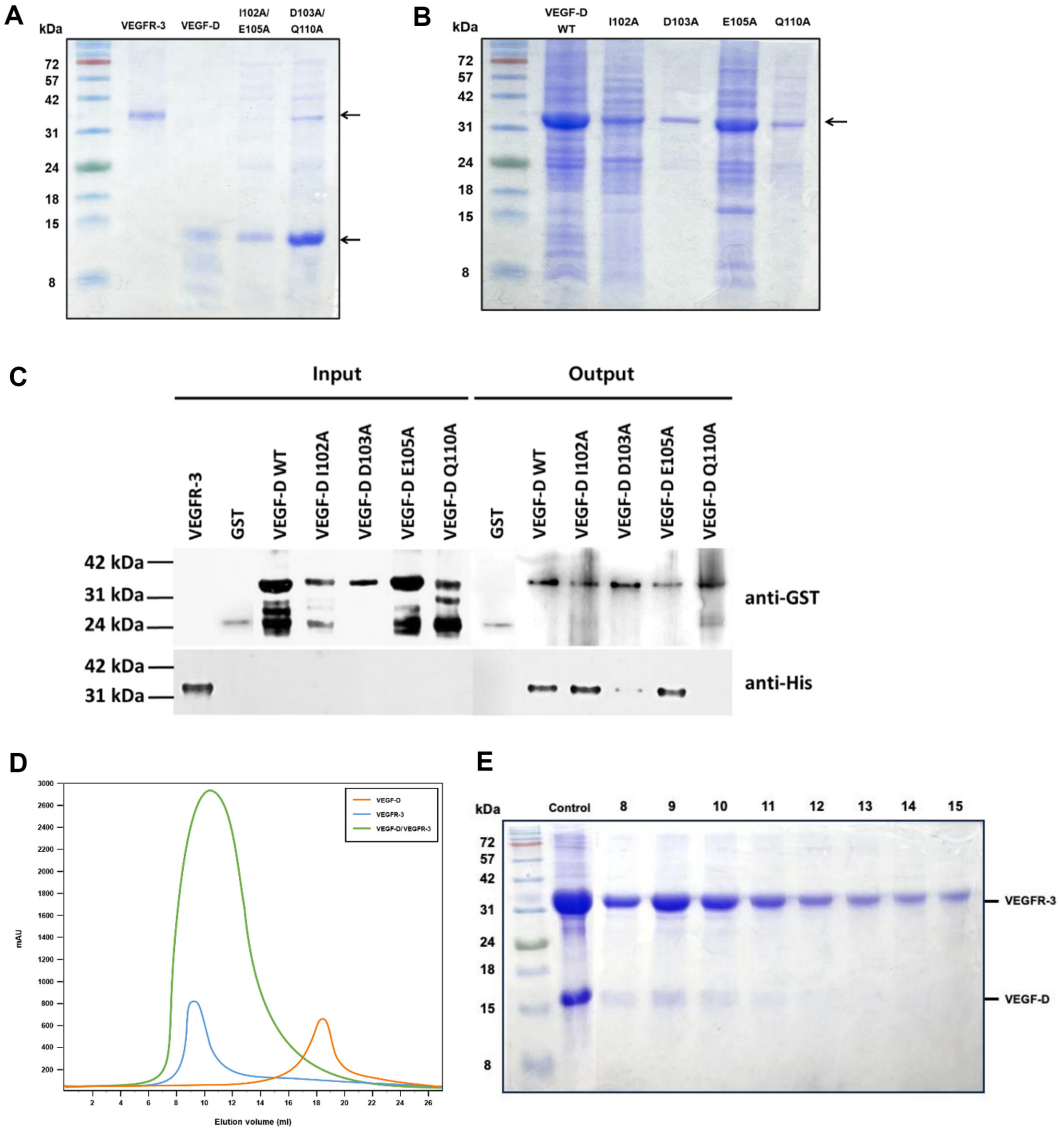
Interaction analysis between VEGF-D and VEGFR-3. (**A**) The purified His-tagged double mutated form of VEGF-D (I102A/ E105A and D103A/ Q110A) are visualized by SDS-PAGE and Coomassie blue staining. (**B**) The purified GST-tagged wild-type VEGF-D and mutants (I102A, D103A, E105A and Q110A) are visualized by SDS-PAGE and Coomassie blue staining. (**C**) A GST pull-down assay was performed using GST-tagged wild-type VEGF-D, GST-tagged mutated VEGFD, and His-tagged VEGFR-3. Both wild-type and mutated VEGF-D exhibit a size of approximately 39 kDa, while VEGFR-3 has a size of 35 kDa. The bands were detected using anti-His antibody for VEGFR-3 and anti-GST antibody for both wild-type and mutated VEGF-D. (**D**) Size-exclusion chromatography (SEC) is conducted with VEGF-D/VEGFR-3 complex. Before the SEC experiment, the complex incubated for 24 h at 4°C. In an experiment using only VEGF-D as a sample (orange), a peak appears at 17. In case of VEGFR-3 (blue), a peak appears at 8. VEGF-D and VEGFR-3 protein from co-lystae sample (green) were eluted at 7 to 25. (**E**) After application of SEC, the binding of VEGF-D and VEGFR-3 was revealed using SDS-PAGE. The size of VEGF-D is supposed as 15 kDa, VEGFR-3 as 35 kDa. Proteins in eluted buffer were visualized by SDS-PAGE and Coomassie blue staining.

For identify a clearer understanding of the experiments protein-protein interaction experiment, the recombinant VEGF-D (residues 89-205) and VEGFR-3 (residues 30-326) were expressed in *E. coli* BL21 (DE3). The overexpressed proteins were purified using affinity chromatography and FPLC. The sizes of the final purified His-tagged VEGF-D and VEGFR-3 were predicted as roughly 14 and 35 kDa, respectively (Fig. 3A). The purified His-tagged double mutated form of VEGF-D (I102A/ E105A and D103A/ Q110A) are visualized by SDS-PAGE and Coomassie blue staining (Fig. 3A). The purified GST-tagged wild-type VEGF-D and mutants (I102A, D103A, E105A and Q110A) are visualized by SDS-PAGE and Coomassie blue staining (Fig. 3B). To identify which residue is more important for interaction with VEGFR-3, GST pull-down assay was performed with GST tagged VEGF-D and variants of I102A, E105A D103A and Q110A (Fig. 3C). The different affinity of Each protein is visualized through Western blot analysis. The bands of I102A and E105A are similar to the wild-type, while those of D103A and Q110A are remarkably weak.

### Interaction between VEGF-D and VEGFR-3

The binding of recombinant VEGF-D and VEGFR-3 was detected by size-exclusion chromatography analysis. The His-tagged VEGF-D (residues 89-205) and His-tagged VEGFR-3 (residues 30-326) exhibited the predicted sizes of 15 and 35 kDa, respectively. The peak of VEGF-D/VEGFR-3 complex was shown to have the molecular mass of a 1:1 complex (Fig. 3D and 3E). In this study, the contribution of residues was compared through some experiments. In size exclusion chromatography, it was confirmed that recombinant VEGF-D and VEGFR-3 bind with each other. As a result of the complex formation of VEGF-D and VEGFR-3, the VEGF-D/VEGFR-3 graph begins to rise when elution volume reaches 7 ml (Fig. 3D). Whereas the graph that uses only VEGFR-3 increases at 8 ml. This forward movement of the peak is conjectured that due to the increase in size as VEGF-D binds to VEGFR-3. Moreover, a VEGF-D band in SDS-PAGE is more distinct at 8 ml than 15 ml (Fig. 3E). Meanwhile a peak begins to appear at 16 ml when conducting FPLC with only VEGF-D (Fig. 3D). The early elution of VEGF-D in VEGF-D/ VEGFR-3 sample compared to VEGF-D sample can be attributed to the binding of VEGFR-3 and VEGF-D.

### Decreased Cell Viability as a Consequence of Mutations at Residue D103 and Q110 within VEGF-D in Breast Cancer Cells

**Fig. 4:**
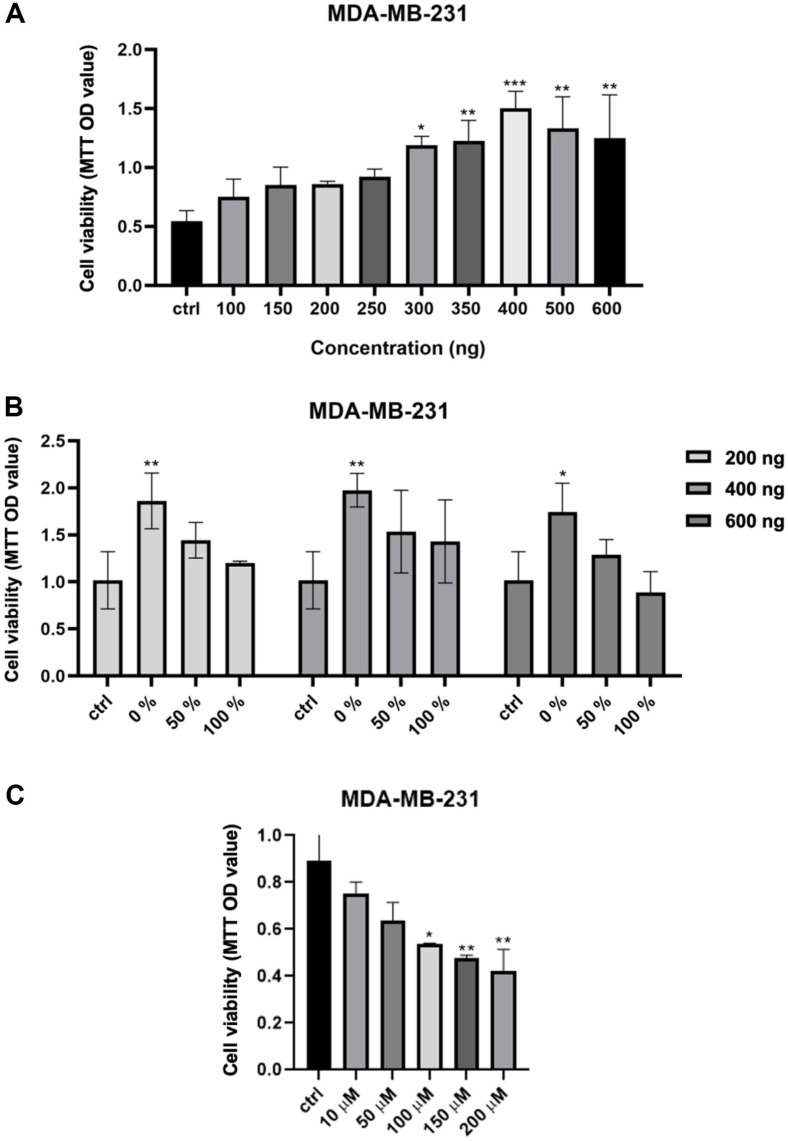
Different bioactive effect of wild-type, mutant, and peptide VEGF-D on cancer cell. (**A, B**) The cell proliferation were examined by MTT assay. Statistic test were performed using one-way ANOVA followed by Bonferroni’s correction. ***P < 0.001, **P < 0.01, *P < 0.05; NS, not significant vs. ctrl. Data are represents as mean ± SD. (**A**) MDA-MB-231 cells were treated with the various concentration (0-600 ng) of VEGF-D for 24 h. Half-maximal effective concentration (EC_50_) value is measured as 238.5 ng. (**B**) Mixtures of wild-type and D103A/ Q110A mutant were treated with the various concentration (0-600 ng) in MDA-MB-231 for 24 h. (**C**) The cell proliferation were examined by MTT assay. Statistic test were performed using one-way ANOVA followed by Bonferroni’s correction. **P < 0.01, *P < 0.05; NS, not significant vs. ctrl. Data are represents as mean ± SD. The MDA-MB-231 cells were treated with the various concentration (0-200 μM) of the VEGF-D peptide for 24 h. Half-maximal inhibitory concentration (IC_50_) value is measured as 49.70 μg.

To investigate whether the wild-type and mutation VEGF-D have different bioactive effects on cancer cells, a cell viability assay was performed (Fig. 4A and 4B). For this, MDA-MB-231, which expresses VEGFR-3 plentifully, was seeded on a 96-well cell culture plate. After seeding, cells were incubated with either wild-type VEGF-D or a mixture of wild-type and D103A/ Q110A mutant for 24 and 48 h. The mixture of wild-type and D103A/Q110A mutants represented the percentage mean mutant ratio versus wild-type in different concentrations (*e.g.*, 0%; none mutant, 50%; half mutant, 100%; all mutant). The wild-type VEGF-D increased cell viability in cancer cells by binding VEGFR-3 (Fig. 4A). However, increasing the D103A/Q110A mutant ratio versus wild-type VEGF-D decreased cell viability on MDA-MB-231 (Fig. 4B). These represented that D103/Q110 residues were significantly correlated as binding with VEGFR-3. The cell viability was increased in a VEGF-D dose-dependent manner. The wild-type VEGF-D exhibited half-maximal effective concentration (EC_50_) value of 238.5 ng.

### Inhibitory Effect of the Peptide on the Viability of Breast Cancer Cells

Based on prediction model of the VEGF-D–VEGFR-3 complex, the peptide was synthesized to target VEGFR-3 as interrupt the binding between VEGF-D and VEGFR-3. To examine the inhibitory effects of the peptide on the VEGFR-3 activity, an in vitro viability assay was performed (Fig. 4C). For this, MDA-MB-231 which express VEGFR-3 plentifully, was seeded on a 48-well cell culture plate. After seeding, the peptide was treated for 24 h. The cell viability was decrease in a peptide dose-dependent manner.

## Discussion

Lymangiogenesis may be encouraged by function of VEGF-D as an activator of VEGFR-3, which downstream signaling regulates cell migration, proliferation, and survival. Furthermore, it has been reported that several cancers are related to lymphangiogenesis. In previous studies, the treatment of a specific antibody for VEGF-D showed its effect on hindrance of lymphatic metastasis [14]. Therefore, it is no exaggeration to say that VEGF-D/VEGFR-3 interaction is weighty in terms of the metastasis and prognosis of cancer patients. Despite their consequence, information about the participation of the VEGF-D residue in VEGFR-3 binding remains unclear. This study aimed to explore the characteristics of binding between VEGF-D and VEGFR-3 and suggests key residues for their interaction. In the case of VEGF-C, there is a report that the key regions for interaction with VEGFR-3 are D123 and Q130 [30]. Based on the likeness between VEGF-D and VEGF-C that is supported by their secondary structure and superimposing figure (Fig. 1D) [11], it was hypothesized that VEGF-D has a crucial residue in locations similar to those in VEGF-C. Unlike this assumption, other studies have reported that other residues including I102 and E105 are essential [31]. In this study, the contribution of residues was compared through some experiments.

The difference between the wild-type and the variants in the affinity to VEGFR-3 is shown in the SPR analysis (Fig. 2A-2C). Strong signals were detected in the wild-type VEGF-D and relatively weak signals were detected in the variant VEGF-D samples. By CD analysis, increase and decrease are more remarkable in D103A/ Q110A mutant. This implies that more structural changes occur when D103 and Q110 are mutated in VEGF-D than I102 and E105. Furthermore, it can be reason that D103 and Q110 are more essential for VEGF-D structure. In GST pull-down assay, it was observed that the bands of D103A and Q110A were relatively weak. Based on this, it can be assumed that D103A and Q110A are more important than I102A and E105A in VEGFR-3 binding (Fig. 3A).

Based on secondary structure alteration and diminution in VEGFR-3 binding of the mutants, it is hypothesized that the D103A/ Q110A mutant of VEGF-D exhibits low effect on VEGFR-3 also in vitro. The viability of breast cancer cell (MDA-MB-231) was analyzed after the treatment of the VEGF-D and mutant for 24 h. A dose-dependent increase in cell viability was observed in cells that were treated with increasing doses of the VEGF-D (Fig. 4A). In the treatment of wild-type and mutant mixture, cell viability decreased in a mutant dose-dependent manner (Fig. 4B). This result can be deemed that the recombinant wild-type VEGF-D stimulates VEGFR-3 activation, and the alanine substitution of D103 and Q110 in VEGF-D reduces the effect on VEGFR-3 activation.

To block the interaction between VEGF-D and VEGFR-3, the peptide was synthesized. The peptide was designed on the basis of the VEGF-D residues that are assumed crucial in VEGFR-3 binding. Through MTT, the effect of peptides on cell proliferation was investigated and cell viability decreased in a VEGF-D peptide dose-dependent manner. The competitive binding of the peptide and VEGF-D to VEGFR-3 diminishes cell proliferation, and it is anticipated that the peptide significantly functions as a metastasis suppressor.

The starting point of this study is the similarity between the structures of VEGF-D and VEGF-C [31]. Even though several studies have been conducted, a three-dimensional structure of VEGF-D/ VEGFR-3 complex has not been revealed. This study could be significant in that it compares VEGF-D and VEGF-C structurally for VEGFR-3 binding.

As a way to block the growth and spread of tumor cell, VEGFR signaling is attracting attention. Anti-lymphangiogenics therapies for cancer cure have yet to show any acute side effects in animal-based studies. This can be combined with conventional chemotherapy and can also be used to reduce the size of primary tumors before surgery [33]. Interrupting the VEGF-D/ VEGFR-3 axis is also expected to block tumor lymphangiogenesis. Finding residues that are involved in VEGFR-3 binding contributes to the development of therapeutic peptide specific for VEGF-D and VEGFR-3 interaction. These peptides might block tumor lymphangiogenesis and lymph remodeling in many human cancers that can cause lymphatic metastasis. Furthermore, the discovery of these residues can contribute to promoting lymphangiogenesis and lymphatic remodeling for treating in lymphoma or inflammatory conditions. As with other peptide drugs, these peptides may contribute to the development of drugs with low immunogenicity and high affinity and specificity at low production costs.

## Supplemental Materials

Supplementary data for this paper are available on-line only at http://jmb.or.kr.
