# Supplementary material for: Molecular Characterization and Interaction between Human VEGF-D and VEGFR-3
Source: J Microbiol Biotechnol. 2024 Nov 20;34(12):2627–36. doi: 10.4014/jmb.2409.09060 (PMC11729338; doi:10.4014/jmb.2409.09060)
Supplement: Supplementary file 1 [file jmb-34-12-2627-supple.pdf]

J. Microbiol. Biotechnol. 2024. 34: 2627–2636
https://doi.org/10.4014/jmb.2409.09060

Supplementary Tables and Figures

Molecular Characterization and Interaction between Human VEGF-D and Human VEGFR-3

Chae Eun Seo, Han Na Lee, Mi Suk Jeong and Se Bok Jang*

[1]Department of Molecular Biology, College of Natural Sciences, Pusan National University, Busan 46241, Republic of Korea

[2]Insitute of Systems Biology, Pusan National University, Busan 46241, Republic of Korea

Table S1. Primer sequences of VEGF-D, mutated VEGF-D and VEGFR-3.

Protein			Sequence		Enzyme
VEGF-D	F	5'	CGGGATCCGCGGCAACTTTCTATGAC	3'	*BamHI*
	R	5'	CCGCTCGAGTTAGGATCTTCTGATAATTGA	3'	*XhoI*
I102A	F	5'	AACACTAAAAGTT**GCT**GATGAAGAATGGC	3'	
	R	5'	GCCATTCTTCATC**AGC**AACTTTTAGTGTT	3'	
D103A	F	5'	ACTAAAAGTTATA**GCT**GAAGAATGGCAAA	3'	
	R	5'	TTTGCCATTCTTC**AGC**TATAACTTTTAGT	3'	
E105A	F	5'	AGTTATAGATGAA**GCT**TGGCAAAGAACTC	3'	
	R	5'	GAGTTCTTTGCCA**AGC**TTCATCTATAACT	3'	
Q110A	F	5'	ATGGCAAAGAACT**GCT**TGCAGCCCTAGAG	3'	
	R	5'	CTCTAGGGCTGCA**AGC**AGTTCTTTGCCAT	3'	
I102A& E105A	F	5'	AACACTAAAAGTT**GCT**GATGAA**GCT**TGGC	3'	
	R	5'	GCCA**AGC**TTCATC**AGC**AACTTTTAGTGTT	3'	
VEGFR-3	F	5'	GGAATTCCATATGCCGACCTTGAACATCACG	3'	*NdeI*
	R	5'	CCCAAGCTTAATGACCTCGGTGCTCTC	3'	*HindIII*

Underlines denote restriction enzyme site. Boldfaces denote a mutation site.

Table S2. Binding residues and interaction distance in the prediction model.

VEGF-D	VEGFR-3	Interaction Distance (Å)
O (I96)	OG1 (T168)	3.14
OD1 (D103)	NE1 (W177)	2.77
OD1 (D103)	O (W177)	3.03
OD2 (D103)	O (P178)	2.69

Table S3. Surface plasmon resonance response analysis.

	ka (1/Ms)	kd (1/s)	Kd (M)
**Wild-type VEGF-D**	4314	0.001154	2.676×10^{7}
**I102A/ E105A**	1569	0.0015	9.560×10^{7}
**D103A/ Q110A**	1940	0.001035	5.333×10^{7}

Table S4. Secondary structure proportion from circular dichroism analysis.

	Wild-type VEGF-D	I102A/E105A	D103A/Q110A	Wild-type VEGFR-3
**α-helix**	10	8	7	0
**β-sheet**	20	29	36	43
**Turn**	4	5	3	6
**Random coil**	68	58	54	51

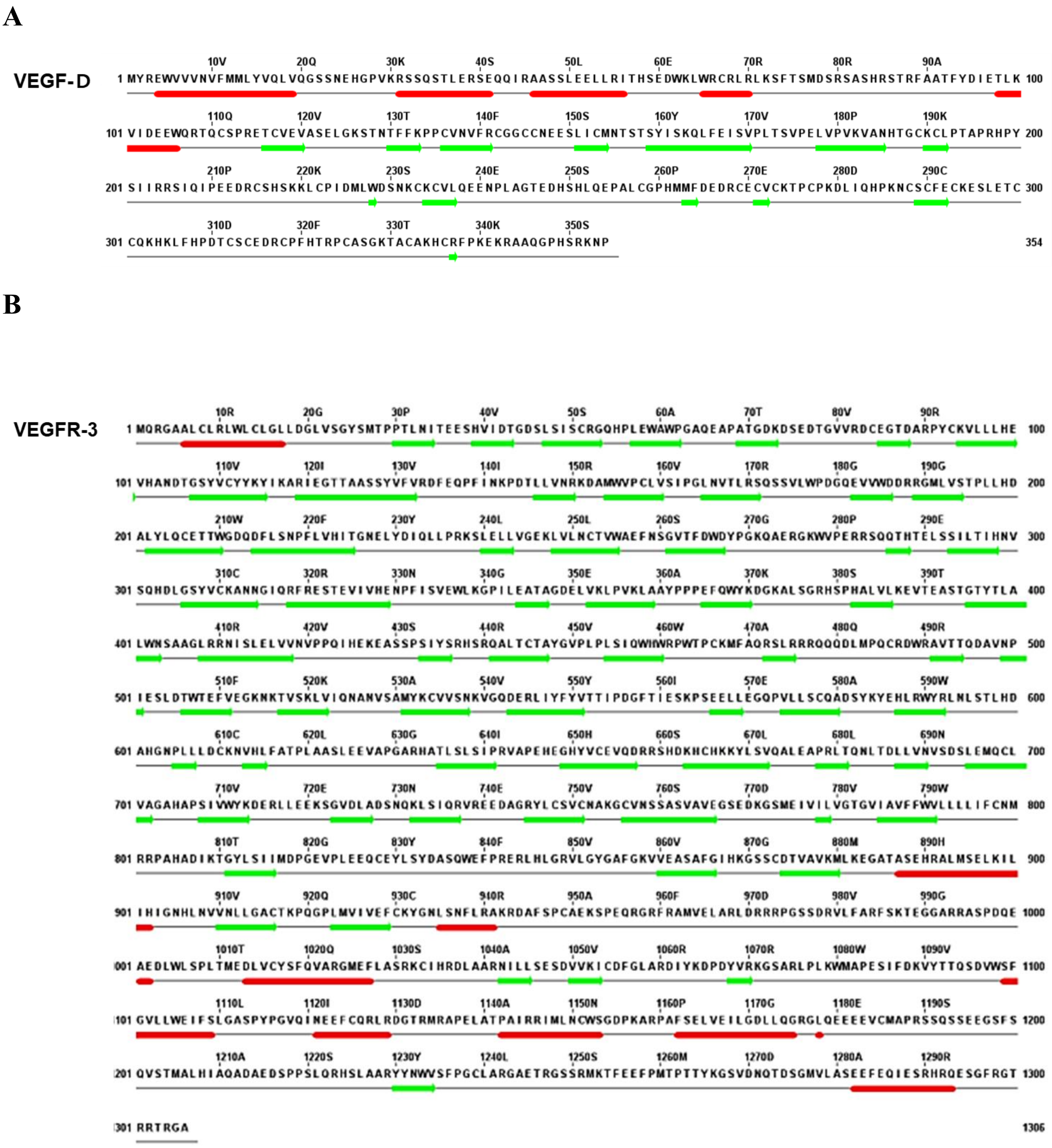

Fig. S1. Secondary structures of VEGF-D and VEGFR-3. (A-B) The sequences and secondary structure of VEGF-D **(A)** and VEGFR-3 **(B)** are created using Jalview program. Alpha-helices are marked as red tubes, beta-sheets as green arrows and loops as line.

A

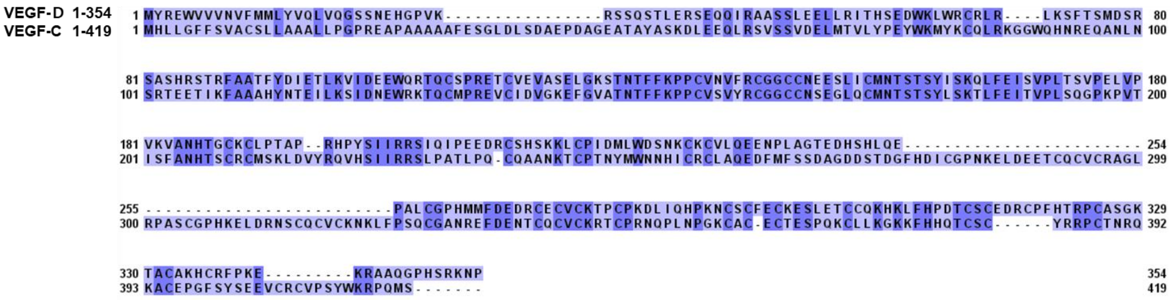

B

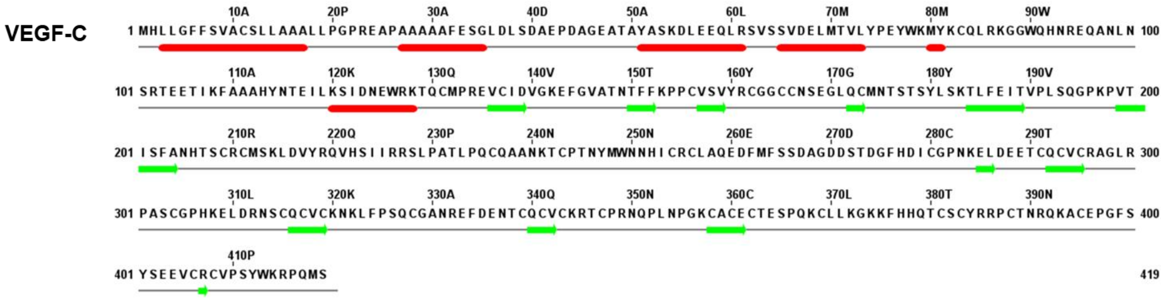

Fig. S2. Alignment between VEGF-D and VEGF-C. (A) VEGF-D protein sequence is aligned with VEGF-C protein sequence. The figure was prepared using Jalview. **(B)** The sequences and secondary structure of VEGF- C is created using Jalview. Alpha-helices are marked as red tubes, beta-sheets as green arrows and loops as line.
